# Ethical and Quality of Care-Related Challenges of Digital Health Twins in Older Care Settings: Protocol for a Scoping Review

**DOI:** 10.2196/51153

**Published:** 2024-02-23

**Authors:** Md Shafiqur Rahman Jabin, Emillia Vann Yaroson, Adaobi Ilodibe, Tillal Eldabi

**Affiliations:** 1 Department of Medicine and Optometry Linnaeus University Kalmar Sweden; 2 Faculty of Health Studies University of Bradford Bradford United Kingdom; 3 Department of Operations and Analytics University of Huddersfield Huddersfield United Kingdom; 4 Department of Applied Artificial Intelligence and Data Analytics University of Bradford Bradford United Kingdom; 5 Faculty of Management, Law & Social Sciences University of Bradford Bradford United Kingdom

**Keywords:** accessibility, data security, effectiveness, equality, health equity, patient safety, right to privacy, social care

## Abstract

**Background:**

Digital health twins (DHTs) have been evolving with their diverse applications in medicine, specifically in older care settings, with the increasing demands of older adults. DHTs have already contributed to improving the quality of dementia and trauma care, cardiac treatment, and health care services for older individuals. Despite its many benefits, the optimum implementation of DHTs has faced several challenges associated with ethical issues, quality of care, management and leadership, and design considerations in older care settings. Since the need for such care is continuously rising and there is evident potential for DHTs to meet those needs, this review aims to map key concepts to address the gaps in the research knowledge to improve DHT implementation.

**Objective:**

The review aims to compile and synthesize the best available evidence regarding the problems encountered by older adults and care providers associated with the application of DHTs. The synthesis will collate the evidence of the issues associated with quality of care, the ethical implications of DHTs, and the strategies undertaken to overcome those challenges in older care settings.

**Methods:**

The review will follow the Joanna Briggs Institute (JBI) methodology. The published studies will be searched through CINAHL, MEDLINE, JBI, and Web of Science, and the unpublished studies through Mednar, Trove, OCLC WorldCat, and Dissertations and Theses. Studies published in English from 2002 will be considered. This review will include studies of older individuals (aged 65 years or older) undergoing care delivery associated with DHTs and their respective care providers. The concept will include the application of the technology, and the context will involve studies based on the older care setting. A broad scope of evidence, including quantitative, qualitative, text and opinion studies, will be considered. A total of 2 independent reviewers will screen the titles and abstracts and then review the full text. Data will be extracted from the included studies using a data extraction tool developed for this study.

**Results:**

The results will be presented in a PRISMA-ScR (Preferred Reporting Items for Systematic Review and Meta-Analysis extension for Scoping Reviews) flow diagram. A draft charting table will be developed as a data extraction tool. The results will be presented as a “map” of the data in a logical, diagrammatic, or tabular form in a descriptive format.

**Conclusions:**

The evidence synthesis is expected to uncover the shreds of evidence required to address the ethical and care quality-related challenges associated with applying DHTs. A synthesis of various strategies used to overcome identified challenges will provide more prospects for adopting them elsewhere and create a resource allocation model for older individuals.

**International Registered Report Identifier (IRRID):**

DERR1-10.2196/51153

## Introduction

### Overview

Digital health twins (DHTs), defined as digital representations (digital twins) of patients (physical twins), are an emerging technology aimed at meeting some of the above challenges. DHTs are generated from multimodal patient data, population data, and real-time updates on patient and environmental variables [1]. The field of a digital twin, particularly in health care, has been evolving by using machine learning data aggregated from various patients through modeling the conditions and attributes of a particular patient [2].

The application of digital twins in health and medicine is diverse; for example, DHTs have been used in intensive care units for critical care [3], treatment of patients with trauma [4], and cardiac treatment [5]. Precision health or personalized care for primary prevention with DHTs for older adults has emerged to address the need to mitigate these significant resources and reinforcements. This can allow for collecting data that enables more individually tailored interventions, both to promote and prevent, diagnose, and treat—interventions with greater precision [6].

Moreover, digital twins have widely been used in older care settings, for example, care homes [7] or for older individuals in different settings, such as hospitals [8]. The evidence suggests that the application of DHTs in dementia is broad, such as detecting the signs of dementia [8], assessing the risks associated with Alzheimer disease–related dementia [9], and supporting better dementia care [10]. DHTs have also been found to improve health care services for older individuals by providing real-time supervision and accurate crisis warning [11] and to improve the efficiency and accessibility of medical services for older individuals [12].

With the increase in life expectancy, older adults are growing in the United Kingdom and around the world and are expected to live longer with multiple disabilities and diseases [13]. Around 19% of the population in the United Kingdom is aged 65 years or older, many of whom require health and social care services to enable them to live longer and have a better quality of life [14]. This number is expected to grow by a further 77% (from 1.4 million to 2.4 million) for older adults by 2040 [15]. While this demographic shift makes older adults the major users of health and care resources [16], it places an unprecedented strain on the workforce of the social care sector [17].

Health and social care for older adults is a complex sociotechnical system that undergoes continuous change [18]. Over the last 3 decades, quality of care has progressively been on the agenda because the care delivery can itself harm patients [19,20], either by providing less than optimal care [21] or by things going wrong with its delivery [22,23]. Whenever a new technology or technological innovation is introduced to improve the quality of care, such as efficiency and effectiveness, new and often unforeseen issues emerge that require attention and awareness [18,21,24]. Thus, new challenges arise continuously, which need resetting priorities for other dimensions of quality of care [18], such as patient safety [25] and health equity [26]. This new technology, that is, the implementation of DHT, is no exception, which can even cause technical [27], ethical [26,27], legal [27,28], and societal challenges [27].

The challenges associated with management and leadership include a lack of discussions, cooperation, negotiations, and agreement among various health and social care providers while new routines are being adopted [6,29]. Design considerations concerning big data-related problems involve data visualization, data availability and accessibility, data integration and interoperability into clinical workflow [10], and privacy and security across the entire system [6,10]. Issues involving the quality of care in terms of DHT implementation are safety, equity, and appropriateness [10,26,28]. Some of the ethical challenges regarding the application of DHTs include autonomy, informed consent, the right to privacy, and surveillance health care [26].

The UK Government’s Plan for Tech and Digital Economy desires an expansion of the United Kingdom’s existing expertise in deep foundational technologies, including digital twins and artificial intelligence [30]. This has been supported by the Government’s Plan for Digital Health and Social Care to expedite technology adoption [31], while the UK Research and Innovation (UKRI) inaugurated a funding scheme to establish a multidisciplinary research community in digital twinning [32]. The UKRI’s initiative focuses on addressing aspects of ethics, human interaction, environmental sustainability and security, and resilience [32], while NHS (National Health Service) England emphasizes the digital transformation of adult social care—the need for personalized care [33].

In order to shape this review, we will focus on older individuals and consider the following dimensions for the quality of care: safety, equity, effectiveness, and accessibility. Conducting a scoping review for the care of older adults is the most ideal and reliable approach to illuminate the current issues associated with DHTs. There is a need for mapping the key concepts to address questions beyond those associated with the experience and effectiveness of this intervention due to DHT’s evolving applications in older care settings [34]. Our proposed review is in line with the vision of the NHS and the UKRI to understand the challenges faced by health and social care professionals, older adults, and their relatives concerning DHT used in personalized care for older adults. Therefore, the review results can readily be accepted, with more prospects of being adopted elsewhere and meeting today’s societal challenges—creating a model for gathering individualized data, disease prevention, monitoring, and resource allocation for older adults.

A preliminary search of Campbell systematic reviews, the Cochrane Database of Systematic Reviews, PROSPERO (International Prospective Register of Systematic Reviews), and Joanna Briggs Institute (JBI) Evidence Synthesis was conducted; however, no current or underway systematic reviews or scoping reviews on the topic were identified.

### Aim and Review Questions

The primary purpose of this scoping review is to compile and synthesize the best available evidence regarding the challenges of DHT encountered in older care settings. This review is believed to uncover the shreds of evidence that will require diligent attention to address the ethical challenges and the challenges concerning the quality of care associated with the application of DHT. Moreover, this study will identify the strategies that have been used to overcome those challenges in older care settings. Therefore, there is a need for new knowledge through evidence synthesis using existing knowledge to be put to good use.

Specifically, the review questions are as follows:

1. What problems are faced by older individuals (their family and relatives) and health and social care providers associated with the application of DHTs in older care settings?
2. What are the documented issues related to the quality of care for older adults, such as safety, equity, effectiveness, and accessibility, concerning DHTs?
3. What are the ethical challenges concerning the application of DHTs in older care settings?
4. What strategies have been evaluated and implemented in older care settings that address the challenges associated with DHTs?

## Methods

The proposed scoping review will be conducted in accordance with the JBI methodology for scoping reviews [35].

### Search Strategy

Databases will be searched for both published and unpublished studies. The approach to searching for studies for a scoping review will follow the standard 3-step method (Table 1). The first step will be an initial limited search of a selection of relevant databases, followed by an analysis of text words in the title and abstract and the index terms used to describe the article. The search for published studies will include a 2-way search strategy. One is to search the journal and reference databases, such as CINAHL, MEDLINE, JBI, and Web of Science. Another is to search article-based (journal) databases, such as the ACM digital library, IEEE Xplore, and BMJ Journals. The search for unpublished studies will include Mednar, Trove, OCLC WorldCat, and Dissertations and Theses. A second search using all identified keywords and index terms will then be undertaken across all included databases. Additional search strategies, that is, citation search—specific researcher or article (eg, gold-standard article), and chain search—review reference list of the systematically selected articles will be included to complement the search for published and unpublished papers. Studies, such as reviews (systematic, scoping, and umbrella) and letters to editors, will be excluded. Any studies that lack ethical concerns will also be excluded. Studies published in English will be considered. Studies published from 2002 (when the concept of “digital twin” was first coined by Dr Michael in 2002) [36] onward will be considered for inclusion in this review.

**Table 1:** Search strategy on databases.

Participant, concept, and context scheme	#	Search string	Hits on MEDLINE (July 18)	Hits on CINAHL (July 18), n	Hits on APA PsycInfo (July 18), n	Hits on Web of Science (July 18), n	Hits on Scopus (July 19), n	Hits on WorldCat (July 20), n	Hits on JBI^a^ (July 20), n
Digital health twin in older care settings	1	“digital health twin*” OR “digital twin*” OR “digital phenotype” OR “digital shadow” OR “virtual patient*” OR “personalised health model*” OR “digital patient model” OR “in silico patient*”	6,404,833	639	2982	11,763	17,051	36,108	2
Digital health twin in older care settings	2	older OR aged OR elderly OR senior* OR elder OR “old person*” OR “older person*” OR “old people” OR “older adult*” OR “older people” OR geriatric*	2457	1,251,839	829,113	5,690,683	6,817,585	31,208,688	1773
	3	# 1 AND #2	204	46	21	449	327	1682	0
Health care setting	4	health* OR hospital* OR care* OR caring OR nursing OR treatment OR aid OR management OR therapy	18,297,577	5,152,991	2,786,771	21,428,220	23,457,440	5180	2837
Combined	5	#3 AND #4	189	43	19	308	250	1384	0
Filters	6	#3 + English, from 2002 onward (exc meeting abstract, editorial material and book chapters.	197	45	21	428	309	8	0
Filters	7	#5 + English, from 2002 onward (exc meeting abstract, editorial material and book chapters.	184	43	19	300	237	4	0

^a^JBI: Joanna Briggs Institute.

### Eligibility Criteria

#### Overview

This scoping review will include the following PCC mnemonics—population, concept, and context. These mnemonics will be used as a guide (not a policy); therefore, the inclusion criteria of this systematic review will include a detailed description of types of participants, concepts, and context, as well as search strategies, data extraction, charting, analysis, and presenting the results. The eligibility criteria are listed in Textbox 1.

**Inclusion and exclusion criteria.**

**Inclusion criteria**

- Review articles
- Conference paper
- Gray literature
- Early access
- English
- Studies involving older individuals associated with a digital health twin (DHT), regardless of gender, age, ethnicity, socioeconomic status, disorders, or disability.
- Studies that include paid or unpaid carers, whether they are family members or friends.
- Studies that include care providers involved in older care settings and DHTs, whether they are licensed or unlicensed.
- Studies that evaluate and discuss the process and application of DHTs involving caregivers, older individuals, or family, friends, or relatives.
- Studies from 2002 through 2023.
- Studies that specifically evaluate and discuss the process and application of DHTs in settings such as geriatric wards of primary health care, hospitals or clinics, nursing homes, care homes, and home care facilities for older individuals.

**Exclusion criteria**

- Meeting abstracts
- Editorial materials
- Book chapters
- All other languages
- Studies that do not involve older individuals or care providers associated with DHTs in older care settings.
- Studies that do not focus on the application of DHTs or are not directly related to older care settings.
- Studies from 2001 and earlier.
- Studies not in the context of older care settings.
- Studies where DHTs are not used.

#### Participants

This review will include studies of older individuals (aged 65 years or older) undergoing care delivery associated with DHTs, irrespective of gender and diversity, including age, ethnicity, socioeconomic status, disorders, and disability. Studies that focus on caregivers (family and friends—paid or unpaid) and care providers (licensed or unlicensed) involved in the care of older adults in relation to DHTs will also be included.

#### Concept

In this scoping review, the key concept is the process and application of DHTs. Studies that evaluate the application of DHTs involving older care providers, older individuals, family, friends, or relatives will be considered.

#### Context

The systematic review will consider studies that are based in the older care settings associated with DHTs, such as geriatric wards of primary health care, hospitals or clinics, old-age homes, nursing homes, care homes, and home care facilities for older individuals.

### Types of Sources

This scoping review will consider both experimental and quasi-experimental study designs, including randomized controlled trials, nonrandomized controlled trials, before and after studies, and interrupted time-series studies. In addition, analytical observational studies, including prospective and retrospective cohort studies, case-control studies, and analytical cross-sectional studies, will be considered for inclusion. This review will also consider descriptive observational study designs, including case series, individual case reports, and descriptive cross-sectional studies, for inclusion.

Qualitative studies that focus on qualitative data, including but not limited to designs such as phenomenology, grounded theory, ethnography, qualitative description, and action research, will also be considered.

Text and opinion papers regarding the benefits and challenges of DHT and strategies to overcome the challenges posed by DHTs will also be considered as a scoping review that includes a broad scope of evidence.

### Study or Source of Evidence Selection

Following the search, all identified citations will be collated and uploaded into EndNote (version 20; Clarivate Analytics), and duplicates will be removed. Following a pilot test, titles and abstracts will then be screened by 2 or more independent reviewers for assessment against the inclusion criteria for the review. Potentially relevant sources will be retrieved in full, and their citation details will be imported into the JBI System for the Unified Management, Assessment and Review of Information [37]. The full text of selected citations will be assessed in detail against the inclusion criteria by 2 or more independent reviewers. Reasons for the exclusion of sources of evidence in full text that do not meet the inclusion criteria will be recorded and reported in the scoping review. Any disagreements that arise between the reviewers at each stage of the selection process will be resolved through discussion or with an additional reviewer or reviewers. The results of the search and the study inclusion process will be reported in full in the final scoping review and presented in a PRISMA-ScR (Preferred Reporting Items for Systematic Reviews and Meta-Analyses extension for Scoping Reviews) flow diagram [38].

### Data Extraction

Data will be extracted from papers included in the scoping review by 2 or more independent reviewers using a data extraction tool developed by the reviewers. The data extracted will include specific details about the participants, concept, context, study methods, and key findings relevant to the review questions.

A draft charting table will be developed as a data extraction tool. The charting table will be modified and revised as necessary during the process of extracting data from each included evidence source. Modifications will be detailed in the scoping review. Any disagreements that arise between the reviewers will be resolved through discussion or with an additional reviewer or reviewers. If appropriate, authors of papers will be contacted to request missing or additional data, where required.

## Results

The results will be presented as a “map” of the data extracted from the included papers in a logical, diagrammatic, or tabular (as necessary) form and in a descriptive format that aligns with the objective and scope of the review. Some critical information that the charting table will include is (but is not limited to): year of publication, country of origin for the study, aims, study population and sample size, methodology or methods, type of intervention or comparator, duration of the intervention, and type of outcomes and how they were measured (if applicable). A clear explanation for each category will be provided, accompanied by a narrative summary describing how the results relate to the review objective and questions.

This scoping review protocol was first developed by the principal author as part of a postdoctoral fellowship at Linnaeus University in February 2022. Later, the protocol was refined and continued as part of research development at the University of Bradford by the lead author, who has been undertaking a full search since July 2023. We expect the analysis to be completed by January 2024 and the final scoping review manuscript to be submitted by April 2024.

## Discussion

### Overview

The world’s population is getting older—aging brings with it complicated and multiple challenges such as poorer mobility, balance, sight or hearing, dementia, diabetes, and an increased risk of injuries [39]. With the trend of increasing older adults, it is estimated that almost 1 in 6 adults must work in care by 2038 to meet the demand for older adults in home care alone. This is a hard target to meet, given the current shortfalls in the workforce available in social care sectors, which became more apparent during the COVID-19 pandemic [17]. Not to mention the lack of incentive in many countries to take such jobs. Since technology is seen as a means to address the shortage of care workers by health and social care policy makers [13], we believe that DHTs have the capability of addressing the demand for the care of older adults and compensating for the shortage of care workers.

A recent rapid literature review of studies of DHT for managing health care systems identified 17 studies, concluding that DHT can contribute to safety management, information management, health management and well-being promotion, and health care operational control [40]. With appropriate implementation, DHT helps treat patients as virtualized standalone assets [41], improve patient treatment and diagnostics [6], and improve the quality of life overall [42].

The current concept of DHT is diverse as it is still a new and emerging domain, particularly in older care settings [7-10,34], with the aim of improving the quality of care by increasing efficiency [43]. New definitions, concepts, and dimensions in regard to DHT may be identified in this review that may have the potential to help in furthering the design, development, implementation, and evaluation process of DHT. We believe that the evidence synthesis will help us design a larger implementation project, that is, assembling unique and existing technologies to create a new digital twin platform for older care settings.

Since DHTs are expensive and sometimes may not be readily available to older adults to a large extent [44], this study will assess the different circumstances and needs of older adults. This study will ensure appropriate resources and solutions are allocated to needy individuals, covering aspects of equity, and closing the gap in health inequality. The benefits of DHT will help us understand informed decision-making in older care settings.

### Limitations

The findings of the review need to be treated with caution since our search is restricted in terms of language and publication period. To overcome these limitations, a comprehensive strategy, that is, a standard 3-step method, will be followed, such as the inclusion of gray literature, which may provide additional insights into the review findings. There may also be a possibility pertaining to the findings in terms of the limited number of included studies, which may further add a layer of bias to the selected studies. The evidence-based practice center methods guide proposed by the Agency for Healthcare Research and Quality will be followed to minimize the risk of bias in individual studies [45].

To ensure the systematic scoping review generates generalizable findings, discussions with older adults, their relatives, and the relevant public will be held to support the interpretation of the findings and dissemination of the review.

### Conclusions

No current or ongoing systematic reviews or scoping reviews on the topic were identified. This review will uncover the shreds of evidence requiring diligent attention to address the ethical challenges and the challenges concerning the quality of care associated with the application of DHT. Additionally, this study will identify the strategies that have been used to overcome those identified challenges in older care settings, providing more prospects for adopting them elsewhere and creating a model of resource allocation for older individuals.

## Abbreviations

DHT: digital health twin
JBI: Joanna Briggs Institute
NHS: National Health Service
PRISMA-ScR: Preferred Reporting Items for Systematic Reviews and Meta-Analyses extension for Scoping Reviews
PROSPERO: International Prospective Register of Systematic Reviews
UKRI: UK Research and Innovation

## Data Availability

All data generated during this study will be included in the original review article (and its supplementary information files).
